# Operating room-related risk factors for surgical site infection in general surgeries: A retrospective cohort study

**DOI:** 10.1097/MD.0000000000044565

**Published:** 2025-09-12

**Authors:** Shuang Zhu, Xue Yang, Ping Zhang, Chunmei Chen

**Affiliations:** a Operating Room, The Affiliated Traditional Chinese Medicine Hospital, Southwest Medical University, Luzhou, Sichuan, China; b Central Sterile Supply Department, The Affiliated Traditional Chinese Medicine Hospital of Southwest Medical University, Luzhou, Sichuan, China.

**Keywords:** infection control, multivariate logistic regression, operating room, perioperative care, risk factors, surgical site infection

## Abstract

This study aims to identify modifiable operating room-related risk factors for surgical site infection (SSI) using multivariate logistic regression analysis. This retrospective cohort study was conducted at a tertiary hospital between July 2021 and September 2023, enrolling patients who underwent various general surgical procedures and met the predefined inclusion criteria. Clinical, demographic, intraoperative, and postoperative data were collected from medical records. Independent risk factors for SSI were identified via multivariate logistic regression. Among 479 patients, 60 (12.5%) developed SSI. Multivariate analysis identified several independent risk factors: elevated body mass index (OR = 1.12, 95% CI: 1.05–1.19, *P* <.001), diabetes mellitus (OR = 2.10, 95% CI: 1.14–3.88, *P* = .017), emergency surgery (OR = 3.15, 95% CI: 1.58–6.27, *P* = .001), prolonged surgical duration ≥ 2 hours (OR = 2.42, 95% CI: 1.30–4.52, *P* = .005), conventional operating room environment (OR = 2.20, 95% CI: 1.20–4.03, *P* = .011), intraoperative personnel changes (OR = 3.50, 95% CI: 1.85–6.62, *P* <.001), and poor compliance with infection control measures including instrument sterilization (OR = 4.45, 95% CI: 2.13–9.28, *P* <.001) and hand hygiene (OR = 3.18, 95% CI: 1.71–5.93, *P* <.001). Our findings highlight modifiable intraoperative factors associated with SSI, which may inform future preventive strategies. However, prospective validation is warranted to assess their clinical impact. Several modifiable operating room-related factors significantly increase SSI risk. Several modifiable OR-related factors were significantly associated with SSI risk. These insights may inform future preventive efforts, but further prospective validation is needed.

## 1. Introduction

Surgical site infections (SSI) are among the most frequent and challenging complications following surgical procedures, significantly impacting patient outcomes, hospital costs, and healthcare quality worldwide.^[1,2]^ SSI is associated with prolonged hospitalization, increased readmission rates, higher rates of morbidity and mortality, and substantial financial burdens on healthcare systems.^[3,4]^ Despite advancements in surgical techniques, perioperative care, and infection prevention practices, SSI remains prevalent in both developed and developing countries, highlighting the critical need for continuous investigation into modifiable risk factors and effective preventive measures.^[5]^

In this study, we focused on patients undergoing general surgical procedures, including elective and emergency operations across abdominal, soft tissue, and urological domains. Given that SSI rates can vary significantly across surgical specialties, it is important to contextualize our findings within the scope of general surgery. This focus allows for a broader understanding of modifiable intraoperative risk factors that are applicable across a wide range of surgical contexts, rather than being confined to a single specialty such as orthopedics or cardiothoracic surgery.

Numerous studies have identified patient-related factors such as obesity, diabetes mellitus, hypertension, smoking status, and higher American Society of Anesthesiologists (ASA) scores as significant contributors to SSI risk.^[6–8]^ However, increasing evidence suggests that intraoperative environmental factors, particularly operating room characteristics, surgical practice compliance, personnel behaviors, and adherence to sterilization and hygiene protocols, play equally critical roles in SSI development.^[9,10]^ For instance, previous research has indicated that operating room airflow systems, such as laminar airflow versus conventional ventilation systems, directly influence airborne microbial counts, potentially affecting SSI rates.^[11]^ Moreover, intraoperative personnel dynamics, including frequent changes of surgical team members and the total number of personnel present, have also been recognized as important risk determinants due to increased breaches of aseptic techniques.^[12,13]^

Given the multifactorial nature of SSI, understanding and quantifying modifiable intraoperative risk factors through robust statistical analyses is essential for improving surgical outcomes and informing infection prevention strategies. Although individual risk factors have been studied, comprehensive evaluations of operating room-related determinants remain limited.^[14,15]^

Therefore, this study aimed to systematically identify operating room-related risk factors for SSI through a retrospective cohort analysis using multivariate logistic regression to provide evidence that may inform future preventive strategies. We hypothesized that specific modifiable operating room-related factors are independently associated with SSI incidence, and that their identification could improve the understanding of SSI pathogenesis and guide future prevention strategies.

## 2. Methods

### 2.1. Study design

This retrospective cohort study was conducted at a tertiary teaching hospital in Southwest China between July 2021 and September 2023. Patients undergoing general surgical procedures – including abdominal, urological, and soft tissue operations, both elective and emergency – were enrolled. Inclusion criteria required complete medical records, perioperative data, and no preexisting or unrelated concurrent infections. Patients were followed for 30 days postoperatively to monitor the occurrence of SSI, based on CDC diagnostic criteria.

### 2.2. Patient population

A total of 479 surgical patients were included and divided into 2 groups based on the presence or absence of SSI: the SSI group (n = 60) and the non-SSI group (n = 419). SSI diagnosis followed the criteria defined by the Centers for Disease Control and Prevention (CDC). Exclusion criteria were incomplete medical records, a previous history of SSI, and concurrent infections unrelated to the surgical procedure.

### 2.3. Data collection

Patient demographic data, clinical characteristics, surgical details, and perioperative care data were retrospectively collected from medical records. Variables collected included age, gender, body mass index (BMI), diabetes, hypertension, smoking status, ASA score, surgical type, duration and approach (open vs minimally invasive), wound classification, emergency surgery status, drainage use, intraoperative factors (operating room type, personnel count and changes, sterilization compliance), postoperative management (glucose control, antibiotic adherence, timing of drain removal, wound care), ICU admission, and surgical outcomes.

### 2.4. Statistical analysis

Continuous data were expressed as means ± standard deviation and compared using Student *t*-test. Categorical variables were described as numbers (percentages) and analyzed using Chi-square tests or Fisher exact test where appropriate. Variables with a *P*-value <.10 in univariate analysis and those deemed clinically relevant were considered for inclusion in the multivariate logistic regression model. To minimize the risk of overfitting given the limited number of events (60 SSI cases), we restricted the final model to 10 variables based on the events-per-variable (EPV) principle. Variable selection was guided using a backward stepwise approach with Akaike Information Criterion (AIC) optimization. Multicollinearity among candidate variables was assessed using the variance inflation factor, and variables with variance inflation factor >5 were excluded or combined as appropriate. Adjusted odds ratios (ORs) and 95% confidence intervals were calculated. A *P*-value <.05 (2-sided) was considered statistically significant. Model fit was assessed using the Hosmer–Lemeshow test.

### 2.5. Outcome measures

Primary outcome measure was SSI incidence. Secondary outcomes were hospital length of stay, ICU admission, readmission rates, reoperation rates, sepsis incidence, wound dehiscence, extended antibiotic therapy usage, mortality rates, and overall hospital costs.

### 2.6. Software

All statistical analyses were performed using SPSS software (version 25.0, IBM Corp., Armonk).

## 3. Results

### 3.1. Baseline characteristics of patients with and without SSI

Table 1 summarizes the baseline characteristics of all patients and compares those with and without SSI. Significant differences were observed across multiple domains. Patients who developed SSI had higher BMI, a greater prevalence of comorbidities such as diabetes and hypertension, and were more likely to undergo emergency or contaminated procedures. Intraoperative risk factors such as prolonged surgery, increased personnel changes, and open approach were more common among SSI patients. Additionally, lower adherence to infection control practices – including instrument sterilization, hand hygiene, and sterile technique – was significantly associated with increased SSI risk. Postoperative variables such as ICU admission, poor glucose control, and suboptimal antibiotic use also showed notable differences. Full details, including statistical comparisons and subgroup distributions, are provided in Table 1.

**Table 1:** Baseline characteristics of patients with and without SSI.

Variable	All patients (n = 479)	Non-SSI (n = 419)	SSI (n = 60)	*P*-value
Age (year)	51 ± 9	51 ± 8	51 ± 11	.963
Gender (Male, n [%])	323 (67.4%)	278 (66.3%)	45 (75.0%)	.167
BMI (kg/m²)	25.3 ± 3.5	24.9 ± 3.2	28.3 ± 3.1	<.001
Diabetes (n [%])	107 (22.3%)	86 (20.5%)	21 (35.0%)	.004
Hypertension (n [%])	164 (34.2%)	138 (32.9%)	26 (43.3%)	.018
Smoking history (n [%])	140 (29.2%)	120 (28.6%)	20 (33.3%)	.221
ASA score (I–II, n [%])	310 (64.7%)	290 (69.2%)	20 (33.3%)	<.001
ASA score (III–IV, n [%])	125 (26.1%)	100 (23.9%)	25 (41.7%)	<.001
Surgical type (Clean, n [%])	110 (22.9%)	100 (23.9%)	10 (16.7%)	.138
Surgical type (Clean-contaminated, n [%])	260 (54.3%)	240 (57.3%)	20 (33.3%)	.002
Surgical type (Contaminated/Infected, n [%])	60 (12.5%)	50 (11.9%)	10 (16.7%)	.031
Surgical duration (≥2 hours, n [%])	165 (34.4%)	130 (31.0%)	35 (58.3%)	<.001
Surgical approach (Open, n [%])	335 (69.9%)	290 (69.2%)	45 (75.0%)	.331
Use of drainage (n [%])	145 (30.3%)	120 (28.6%)	25 (41.7%)	.049
Emergency surgery (n [%])	60 (12.5%)	40 (9.5%)	20 (33.3%)	<.001
Wound classification (I, n [%])	130 (27.1%)	120 (28.6%)	10 (16.7%)	.041
Wound classification (II, n [%])	230 (48.0%)	210 (50.1%)	20 (33.3%)	.010
Wound classification (III–IV, n [%])	90 (18.8%)	70 (16.7%)	20 (33.3%)	<.001
Operating room type (Laminar flow, n [%])	300 (62.6%)	270 (64.4%)	30 (50.0%)	.038
Operating room type (Conventional, n [%])	145 (30.3%)	120 (28.6%)	25 (41.7%)	.008
Intraoperative personnel count (≥5, n [%])	160 (33.4%)	130 (31.0%)	30 (50.0%)	.005
Intraoperative personnel change (≥1, n [%])	95 (19.8%)	70 (16.7%)	25 (41.7%)	<.001
Instrument sterilization compliance (n [%])	430 (89.8%)	390 (93.1%)	40 (66.7%)	<.001
Hand hygiene adherence (n [%])	405 (84.6%)	370 (88.3%)	35 (58.3%)	<.001
Sterile technique compliance (n [%])	380 (79.3%)	350 (83.5%)	30 (50.0%)	<.001
ICU admission postsurgery (n [%])	60 (12.5%)	40 (9.5%)	20 (33.3%)	.002
Postoperative glucose control (n [%])	390 (81.4%)	350 (83.5%)	40 (66.7%)	.007
Postoperative antibiotic adherence (n [%])	430 (89.8%)	380 (90.7%)	50 (83.3%)	.041
Drain removal timing (Early, n [%])	130 (27.1%)	120 (28.6%)	10 (16.7%)	.013
Postoperative dressing frequency (Adherent, n [%])	365 (76.2%)	330 (78.8%)	35 (58.3%)	.029

ASA = American Society of Anesthesiologists, BMI = body mass index, ICU = intensive care unit, SSI = surgical site infection.

Values are presented as mean ± standard deviation for continuous variables and number (percentage) for categorical variables. *P*-values were calculated using the Student *t* test for continuous variables and the chi-square test or Fisher exact test for categorical variables, as appropriate. *P* <.05 was considered statistically significant.

### 3.2. Multivariate logistic regression analysis of risk factors for SSI

**Figure 1.**
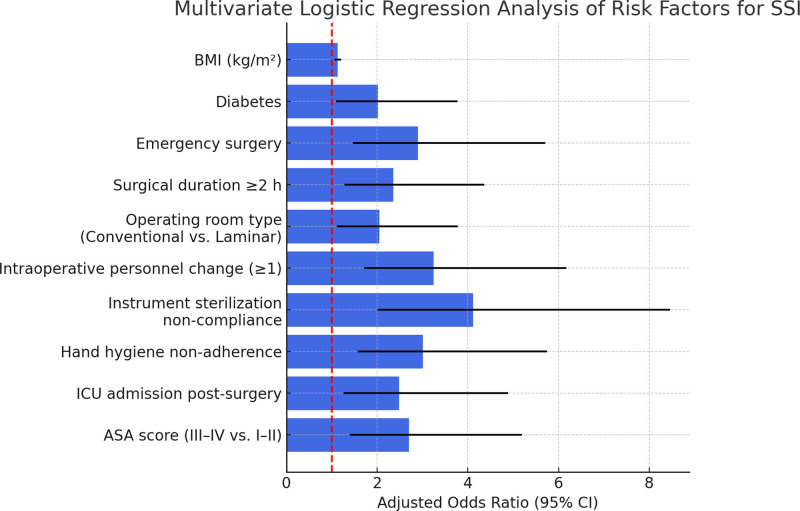
Multivariate logistic regression analysis of independent risk factors for SSI. Bars represent adjusted ORs with 95% CIs. A red dashed line at OR = 1.0 indicates the null value. All variables shown were retained in the final model using backward stepwise selection based on AIC, with multicollinearity assessed using the VIF. The model was adjusted for age and gender. AIC = Akaike information criterion, CIs = confidence intervals, ORs = odds ratios, SSI = surgical site infection, VIF = variance inflation factor.

Table 2 presents the results of the final multivariate logistic regression model, which retained 10 independent predictors of SSI following AIC-based variable selection and multicollinearity adjustment. Higher BMI was significantly associated with increased SSI risk (OR: 1.13, 95% CI: 1.06–1.21, *P* <.001). Patients with diabetes had a 2-fold greater likelihood of developing SSI (OR: 2.02, 95% CI: 1.09–3.77, *P* = .026), and emergency procedures also substantially elevated risk (OR: 2.90, 95% CI: 1.47–5.71, *P* = .002). Surgeries lasting 2 hours or longer were independently associated with higher SSI incidence (OR: 2.36, 95% CI: 1.28–4.36, *P* = .006). Several intraoperative environmental and behavioral factors emerged as significant contributors to SSI. Operations performed in conventional operating rooms, as opposed to laminar flow environments, were associated with a more than 2-fold increase in risk (OR: 2.05, 95% CI: 1.11–3.78, *P* = .022). Intraoperative personnel changes (≥1) were independently predictive of SSI (OR: 3.25, 95% CI: 1.71–6.17, *P* <.001), as were noncompliance with instrument sterilization (OR: 4.12, 95% CI: 2.01–8.46, *P* <.001) and hand hygiene non-adherence (OR: 3.01, 95% CI: 1.57–5.75, *P* <.001). Additionally, patients admitted to the ICU postoperatively demonstrated significantly increased SSI risk (OR: 2.48, 95% CI: 1.26–4.89, *P* = .009). Finally, higher ASA physical status scores (III–IV) were independently associated with infection (OR: 2.70, 95% CI: 1.40–5.19, *P* = .003). These findings emphasize the multifactorial and modifiable nature of SSI risk, highlighting the importance of targeted intraoperative practices, optimal patient selection, and improved perioperative protocols. A graphical representation of the adjusted ORs and confidence intervals for the final model is provided in Figure 1.

**Table 2:** Multivariate logistic regression analysis of risk factors for SSI.

Variable	Adjusted OR (95% CI)	*P*-value
BMI (kg/m²)	1.13 (1.06–1.21)	<.001
Diabetes	2.02 (1.09–3.77)	.026
Emergency surgery	2.90 (1.47–5.71)	.002
Surgical duration ≥ 2 h	2.36 (1.28–4.36)	.006
Operating room type (conventional vs laminar)	2.05 (1.11–3.78)	.022
Intraoperative personnel change (≥1)	3.25 (1.71–6.17)	<.001
Instrument sterilization noncompliance	4.12 (2.01–8.46)	<.001
Hand hygiene non-adherence	3.01 (1.57–5.75)	<.001
ICU admission postsurgery	2.48 (1.26–4.89)	.009
ASA score (III–IV vs I–II)	2.70 (1.40–5.19)	.003

AIC = Akaike information criterion, ASA = American Society of Anesthesiologists, CI = confidence interval, EPV = events-per-variable, ICU = intensive care unit, OR = odds ratio, VIF = variance inflation factor.

Variables included in the final multivariable logistic regression model were selected using backward stepwise elimination based on AIC, constrained by the EPV principle. Variables with VIF > 5 were excluded to reduce multicollinearity. The model was adjusted for age and gender.

### 3.3. Comparison of surgical outcomes between patients with and without SSI

As summarized in Table 3, patients with SSI experienced significantly worse surgical outcomes than those without infection. The SSI group had notably longer hospital stays (15.8 vs 7.2 days, *P* <.001), higher 30-day readmission rates (30.0% vs 2.9%, *P* <.001), and greater postoperative ICU admission rates (41.7% vs 9.5%, *P* <.001). Adverse events such as reoperation (25.0% vs 2.4%, *P* <.001), sepsis (20.0% vs 1.9%, *P* <.001), and wound dehiscence (36.7% vs 3.3%, *P* <.001) were significantly more frequent in the SSI group. Additionally, these patients were more likely to require prolonged antibiotic therapy (91.7% vs 14.3%, *P* <.001) and incurred substantially higher hospital costs (median 25,800 vs 12,500 RMB, *P* <.001). Mortality was also significantly elevated among SSI patients (13.3% vs 1.2%, *P* <.001). These findings underscore the considerable clinical and economic burden associated with SSI and highlight the importance of early identification and preventive strategies in surgical care.

**Table 3:** Comparison of surgical outcomes between patients with and without SSI.

Outcome variable	Non-SSI (n = 419)	SSI (n = 60)	*P*-value
Length of hospital stay (days)	7.2 ± 3.1	15.8 ± 6.4	<.001
Readmission within 30 days (n [%])	12 (2.9%)	18 (30.0%)	<.001
ICU admission postoperatively (n [%])	40 (9.5%)	25 (41.7%)	<.001
Reoperation due to complications (n [%])	10 (2.4%)	15 (25.0%)	<.001
Sepsis occurrence (n [%])	8 (1.9%)	12 (20.0%)	<.001
Wound dehiscence (n [%])	14 (3.3%)	22 (36.7%)	<.001
Extended antibiotic therapy (n [%])	60 (14.3%)	55 (91.7%)	<.001
Mortality (n [%])	5 (1.2%)	8 (13.3%)	<.001
Hospital costs (RMB, median [IQR])	12,500 [10,000–15,500]	25,800 [19,600–32,400]	<.001

ICU = intensive care unit, IQR = interquartile range, RMB = Renminbi (Chinese Yuan), SSI = surgical site infection.

*P*-value derived from *t*-tests, chi-square tests, or Mann–Whitney *U* tests as appropriate.

## 4. Discussion

This study identified several significant operating room-related risk factors contributing to SSIs through a comprehensive multivariate logistic regression analysis. Our final multivariable model identified 10 significant predictors of SSI. Among them, the strongest contributors based on adjusted ORs were instrument sterilization noncompliance (OR: 4.12), intraoperative personnel change (OR: 3.25), hand hygiene non-adherence (OR: 3.01), and emergency surgery (OR: 2.90), highlighting the pivotal role of intraoperative infection control practices and surgical urgency. These findings align with recent studies highlighting that SSI is influenced by a complex interplay of patient, procedural, and environmental factors, emphasizing the need for multifaceted preventive strategies.^[16,17]^

Consistent with previous literature, we found that patient-related comorbidities, particularly obesity and diabetes mellitus, significantly increased SSI risk, likely due to impaired immune responses, delayed wound healing, and increased susceptibility to infections.^[18–20]^ However, our findings uniquely emphasize the critical role of modifiable intraoperative environmental factors. For example, conventional operating room ventilation systems were associated with significantly higher SSI incidence compared to laminar airflow systems. Recent studies have demonstrated that operating room airflow systems directly influence airborne microbial counts and thereby affect infection rates, supporting our observations.^[21,22]^ Our analysis further underscores that intraoperative environmental and behavioral factors, rather than patient-related variables alone, are dominant predictors in the final AIC-optimized model. Furthermore, frequent intraoperative personnel changes and higher personnel counts correlated strongly with increased SSI incidence in our study, echoing prior research indicating that disruptions in surgical team dynamics compromise aseptic protocols and elevate the risk of infection.^[23,24]^

The study’s robust statistical analysis, particularly the use of multivariable logistic regression guided by AIC and constrained by the EPV principle, allowed for precise quantification of the independent effects of each retained predictor, thereby strengthening the validity of our findings. This approach aligns with current recommendations emphasizing evidence-based statistical methodologies to guide infection prevention practices.^[25,26]^ Our findings support the relevance of focusing future preventive efforts on modifiable intraoperative and environmental factors, such as adherence to hygiene protocols and personnel management.

Our findings provide important insights for clinical management and policy-making by identifying modifiable intraoperative risk factors that are associated with increased SSI risk. These results highlight the importance of continued surveillance and surgical quality improvement initiatives aimed at reducing preventable infections.

Nevertheless, our study has several limitations that should be carefully considered when interpreting the findings. Firstly, the retrospective cohort design inherently introduces potential selection bias and confounding variables, as data were collected from existing medical records, limiting control over the completeness and accuracy of documentation. For instance, variations in record-keeping practices among healthcare personnel may have led to incomplete or inconsistent data collection regarding compliance with intraoperative hygiene protocols, sterilization standards, and postoperative wound management. Although comprehensive efforts were made to retrieve complete patient records, missing or inaccurately documented data might have introduced information bias and affected the precision of risk factor estimates. Secondly, the study was conducted in a single tertiary hospital setting, which may limit the generalizability of the findings to other institutions with different patient demographics, surgical specialties, healthcare practices, and operating room environments. Institutional differences in infrastructure, resources, surgical practices, and infection control policies could potentially result in varying effects of identified risk factors and associated infection control practices. Thus, multicenter prospective studies with standardized protocols would be beneficial to validate our findings and further enhance their external validity. Thirdly, although multivariate logistic regression analysis was employed to adjust for confounding variables, residual confounding factors cannot be entirely excluded. Certain potentially influential variables, such as precise antibiotic regimen details, nutritional status, and specific aspects of surgical technique, were not exhaustively analyzed due to limitations inherent in retrospective data availability. Further prospective studies with systematic data collection and detailed control of potential confounders could provide more robust evidence to delineate the relationships among these intraoperative environmental factors and SSI. Additionally, due to the retrospective design and the use of a single-center dataset, potential residual confounding and institutional practice patterns could influence the observed associations. Furthermore, certain intraoperative variables were not captured in sufficient granularity, limiting deeper mechanistic interpretation. Future multicenter prospective studies with standardized data collection protocols are warranted to validate and expand upon these findings. Finally, our observational study design precludes establishing direct causal relationships between identified risk factors and SSI incidence. Despite these limitations, our findings offer valuable insights into operating room-related factors associated with increased SSI risk and provide a foundation for future research. To substantiate these associations and clarify underlying mechanisms, future prospective, multicenter studies – ideally using randomized or rigorously controlled designs – are warranted to support the development of evidence-based infection prevention guidelines.

In conclusion, this study identifies several operating room-related factors that are statistically associated with the occurrence of SSI, providing evidence to support further investigation and potential refinement of infection control strategies. Future prospective, multicenter studies are warranted to confirm these associations and assess the effectiveness of specific preventive measures in reducing SSI incidence.

## Correction

This article was originally published with non-English characters “视双” in section 3.1. The online version has now been updated with the correct sentence **from** “Patients who developed SSI had higher BMI, greater prevalence of comorbidities (e.g., diabetes, 视双), and were more likely to undergo emergency or contaminated procedures.” **to** “Patients who developed SSI had higher BMI, a greater prevalence of comorbidities such as diabetes and hypertension, and were more likely to undergo emergency or contaminated procedures.”

## Author contributions

**Conceptualization:** Shuang Zhu, Xue Yang, Ping Zhang.

**Data curation:** Shuang Zhu, Xue Yang, Ping Zhang, Chunmei Chen.

**Investigation:** Xue Yang, Chunmei Chen.

**Methodology:** Shuang Zhu, Xue Yang, Chunmei Chen.

**Software:** Chunmei Chen.

**Validation:** Ping Zhang, Chunmei Chen.

**Visualization:** Ping Zhang, Chunmei Chen.

**Writing – original draft:** Shuang Zhu, Xue Yang, Ping Zhang, Chunmei Chen.

**Writing – review & editing:** Chunmei Chen.
